# A Method for Real-Time Recognition of Safflower Filaments in Unstructured Environments Using the YOLO-SaFi Model

**DOI:** 10.3390/s24134410

**Published:** 2024-07-08

**Authors:** Bangbang Chen, Feng Ding, Baojian Ma, Liqiang Wang, Shanping Ning

**Affiliations:** 1 School of Mechatronic Engineering, Xi’an Technological University, Xi’an 710021, China; chenbangbang@st.xatu.edu.cn (B.C.); ningshanping@163.com (S.N.); 2 School of Mechatronic Engineering, Xinjiang Institute of Technology, Aksu 843100, China; 2015095@xjit.edu.cn (B.M.); 2019049@xjit.edu.cn (L.W.)

**Keywords:** unstructured environment, safflower filament, lightweight, YOLOv8, target detection

## Abstract

The identification of safflower filament targets and the precise localization of picking points are fundamental prerequisites for achieving automated filament retrieval. In light of challenges such as severe occlusion of targets, low recognition accuracy, and the considerable size of models in unstructured environments, this paper introduces a novel lightweight YOLO-SaFi model. The architectural design of this model features a Backbone layer incorporating the StarNet network; a Neck layer introducing a novel ELC convolution module to refine the C2f module; and a Head layer implementing a new lightweight shared convolution detection head, Detect_EL. Furthermore, the loss function is enhanced by upgrading CIoU to PIoUv2. These enhancements significantly augment the model’s capability to perceive spatial information and facilitate multi-feature fusion, consequently enhancing detection performance and rendering the model more lightweight. Performance evaluations conducted via comparative experiments with the baseline model reveal that YOLO-SaFi achieved a reduction of parameters, computational load, and weight files by 50.0%, 40.7%, and 48.2%, respectively, compared to the YOLOv8 baseline model. Moreover, YOLO-SaFi demonstrated improvements in recall, mean average precision, and detection speed by 1.9%, 0.3%, and 88.4 frames per second, respectively. Finally, the deployment of the YOLO-SaFi model on the Jetson Orin Nano device corroborates the superior performance of the enhanced model, thereby establishing a robust visual detection framework for the advancement of intelligent safflower filament retrieval robots in unstructured environments.

## 1. Introduction

Safflower (*Carthamus tinctorius* L.), a member of the Asteraceae family, is a crop of significant economic importance, utilized for medicinal purposes, oil production, and animal feed [1,2]. It thrives in arid and semi-arid temperate regions. Due to its unique geographical environment, Xinjiang, China, has become the premier base for safflower cultivation, accounting for more than 80% of the national production [3,4]. This plays an invaluable role in promoting regional economic development. However, the unstructured growth of safflower filaments poses significant challenges for mechanized harvesting. Currently, harvesting in Xinjiang primarily relies on manual labor, which is inefficient and leads to reduced activity and yield due to untimely picking [5,6].

In response to this, some domestic universities have developed various types of harvesting machinery, including air-suction [7], comb-clip [8], roller [9], and cutting types [10]. Nevertheless, issues such as filament omission, flower and fruit damage, and excessive manual involvement still persist. These machines still rely on manual assistance and have not achieved efficient, low-damage, automated harvesting.

With the rapid development of artificial intelligence and deep learning technology, research on intelligent agricultural robots has provided a new direction for mechanized safflower filament harvesting. However, the unstructured growth of safflower filaments presents various growth postures and overlaps among filaments, leaves, and flowers. The blooming period and filament color also change with time and light, posing significant challenges for machine vision recognition and picking point location. To address these challenges, Professor Zhang Zhenguo [11] from Xinjiang Agricultural University proposed a rapid detection method for safflower filaments based on an improved YOLOv3. By replacing the original feature extraction network with the lightweight GhostNet and incorporating the CBAM attention mechanism in the Neck, the improved algorithm achieved an average precision of 91.89% and a detection speed of 51.1 frames/s. Professor Guo Hui [12] also proposed a detection and localization algorithm for safflower filaments based on YOLOv5m, improving the Backbone and Neck with CBAM attention mechanism, achieving an accuracy of 95.2% and a model size of 83.72 MB. The team of Wang Xiaorong [13] from Xinjiang University proposed a method for identifying safflower in complex environments based on an improved YOLOv7, optimizing the algorithm by adding the Swin Transformer attention mechanism and improving the Focal Loss function, achieving an average precision of 88.5%, a 7% improvement over the original model. Despite the initial progress, intelligent recognition and harvesting of safflower filaments are still in their early stages. The current research faces challenges such as low recognition accuracy and large model sizes, making it difficult to deploy these algorithms on low-power mobile platforms in the field. Further research is needed on real-time recognition methods for small targets in unstructured growth environments to provide technical support for intelligent safflower filament harvesting robots.

Although the identification of unstructured safflower filaments is still in the exploratory stage, deep learning technology has already been successfully applied in the grading and picking of tea leaves and the identification of small targets. For example, Lu Jun and others [14] proposed a method for detecting tea buds based on an improved YOLOv4-tiny, which introduced the CBAM attention mechanism and BiFPN network to improve detection accuracy while maintaining lightness, achieving a detection accuracy of 97.77%. Mengni Wang [15] from Jiangsu University proposed a tea bud recognition method based on an improved YOLOv5s algorithm, replacing the SPPF structure in the Backbone with ASPP and introducing the CBAM attention mechanism in the Neck to improve the focus on small targets, resulting in a 4.4%, 0.5%, and 4% increase in accuracy, recall, and mean average precision, respectively. Hui Yan and others [16] improved the YOLOv5 model’s ability to recognize small remote sensing targets by incorporating BiFPN and SPD-Conv, achieving a 5.3% higher mAP value than the original model. Sun Hongwei and others [17] proposed a tea leaf visual detection model based on YOLOv8s, combining DCNv2 convolution, GAM attention mechanism, and the SPPFSCP spatial pyramid pooling method, achieving an average precision of 88.27%, a 3.59% improvement over the original model. Yunnan Agricultural University’s Ye Rong [18] proposed a lightweight network YOLOv8-RMDA for early tea disease detection, replacing the C2f module in the Backbone and Neck with RFCBAM and RepGFPN, respectively, and using inner-IoU as the loss function. The improved model achieved an average detection accuracy of 93.04%, 8.15% higher than the base model. In summary, deep learning algorithms have been successfully applied in tea and small target identification, but they are still not mature enough for intelligent safflower filament picking systems. This is mainly due to the unstructured growth of safflower filaments, where most filaments overlap, the blooming period is inconsistent, and there are variations in light conditions. Therefore, it is essential to study a lightweight visual recognition algorithm for small target identification in unstructured environments.

This paper introduces a new algorithm structure YOLO-SaFi based on the improved YOLOv8n model for the rapid real-time detection of small safflower filaments. Firstly, the lightweight StarNet network is introduced into the Backbone network, a new ELC convolution design C2f module is proposed in the Neck network, a new lightweight shared convolution detection head Detect_EL is designed in the Head layer, and the CIoU loss function is improved to PIoUv2. Then, ablation experiments and comparisons with classical visual recognition algorithms were conducted to verify the effectiveness and stability of the improved model. Finally, performance detection in unstructured scenarios, comparison with the baseline model YOLOv8, and deployment on edge devices were performed to further verify the lightweight and reliability of the improved YOLO-SaFi algorithm.

## 2. Materials and Methods

### 2.1. Construction of the Safflower Filament Image Dataset in Unstructured Environments

#### 2.1.1. Safflower Filament Image Collection

To accurately represent the growth state of safflower filaments in unstructured environments, images were collected at the safflower planting base of the Xinjiang Institute of Technology in Aksu, Xinjiang. The planting pattern strictly adhered to the “Technical Specification for Safflower Planting and Collection in Xinjiang” for dryland planting. The safflower plants are relatively short, with most flowers exposed at the top and sides, enabling the robot to capture filament images from these angles for recognition. This configuration also provides sufficient working space for the end effector. The safflower variety used was “Xinjiang Yumin Series Thornless Safflower”.

**Figure 1:**
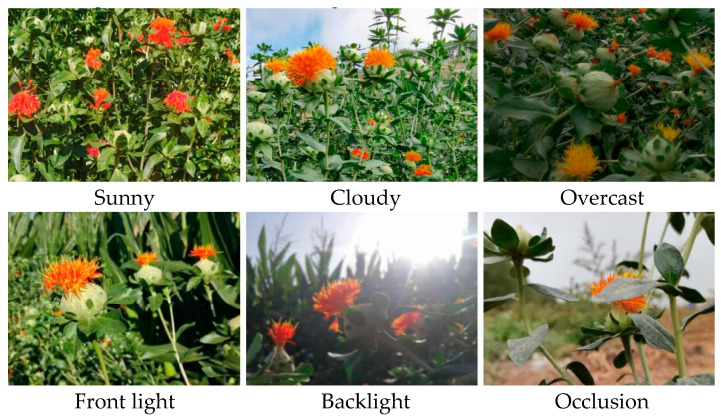
Images of safflower filament in different scenarios.

The safflower filament image acquisition device utilized the Orbbec Gemini 2L camera (Orbbec, Shenzhen, China), featuring a resolution of 1280 × 720 pixels. Images were collected from 7 July to 16 July 2023, during the safflower filament harvesting period, when the height of the safflower plants, as well as the distribution and number of flowers and fruits, were stable. To ensure the diversity of the collected image data, images were taken from multiple angles under three different weather conditions (sunny, cloudy, overcast), two different lighting conditions (front light, backlight), and both obstructed and unobstructed scenes, as depicted in Figure 1. A total of 3000 images were collected and stored in JPG format.

#### 2.1.2. Augmentation and Processing of the Safflower Filament Image Dataset

**Figure 2:**
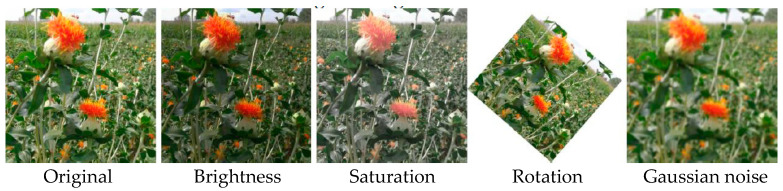
Example of partial data augmentation.

The collected dataset of safflower filament images does not comprehensively cover the variable factors present in unstructured environments. To augment the training set and improve the detection and generalization capabilities of the recognition model, data augmentation strategies such as random brightness, random saturation, random rotation, and random Gaussian noise were applied to the original image data using Python. These techniques aim to encompass as many unstructured environment images as possible. Adjustments in brightness and saturation primarily mimic changes in light intensity due to weather variations encountered during actual recognition, while random rotation and Gaussian blur simulate scenarios caused by robot movement or filament shaking in the wind, as illustrated in Figure 2. These augmentation methods expanded the original dataset to 6500 images, which were divided into training, validation, and test sets in a 7:2:1 ratio, ensuring no duplication to prevent model overfitting. The Labelimg tool was employed to annotate the training and validation datasets. During filament annotation, only the minimum bounding rectangle area of each filament was considered, excluding those with more than 70% occlusion, excessive blur, or those too distant to recognize. Based on safflower filament harvesting stages, the samples were labeled as Safflower-B (blooming period) and Safflower-D (decay period) and saved in XML format to provide a foundational dataset for the intelligent recognition model of safflower filaments.

### 2.2. Safflower Filament Detection Model Based on YOLO-SaFi

**Figure 3:**
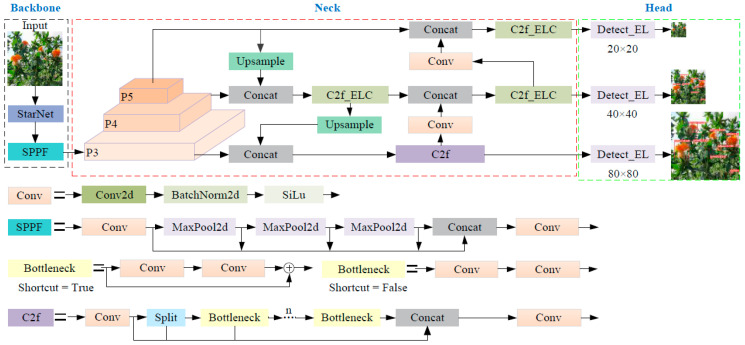
Network structure diagram of YOLO-SaFi.

The real-time detection model for safflower filaments in unstructured environments is primarily deployed on edge devices with limited computing power in the field. This deployment necessitates a model with minimal parameters, rapid recognition speed, and high recognition accuracy. Among the YOLOv8 series [19,20], the YOLOv8n model is the most compact, thus meeting the application requirements for real-time identification and harvesting of safflower filaments. However, the unstructured growth patterns of safflower filaments still present challenges such as missed detections and slow recognition speeds. Consequently, this study proposes an enhanced lightweight algorithm, YOLO-SaFi, based on YOLOv8n. The network structure of this algorithm, depicted in Figure 3, comprises three main layers: Backbone, Neck, and Head. To achieve high detection performance with low computational cost, the Backbone layer integrates the StarNet network [21] to replace the original network for feature extraction. The Neck layer employs the PAN-FPN design concept and introduces the Efficient Lightweight Convolution (ELC) to optimize the C2f module. The Head layer incorporates shared convolution and group normalization to design a lightweight detection head, Detect_EL (Efficient Lightweight). Finally, the PIoUv2 loss function [22] is introduced to address the poor robustness and slow convergence speed of the CIoU.

#### 2.2.1. StarNet Lightweight Network

**Figure 4:**
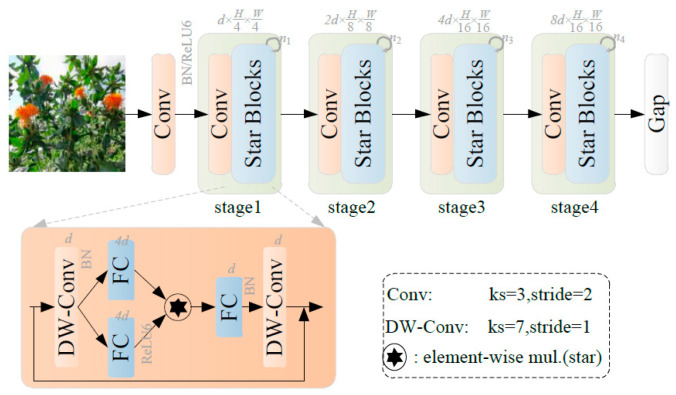
Structure diagram of StarNet.

The YOLOv8 Backbone network employs the DarkNet5 architecture for hierarchical feature extraction, utilizing its C2f module, which incorporates additional Bottleneck structures. This enhances both channel information and computational complexity while extracting richer feature information. To facilitate lightweight deployment on edge devices and efficiently identify safflower filaments in the field for subsequent harvesting, this study introduces the more efficient StarNet network for feature extraction. StarNet outperforms MobileNet [23], ShuffleNet [24], GhostNet [25], FasterNet [26], and EfficientNet [27]. It performs computations in low-dimensional space while generating high-dimensional features, as illustrated in Figure 4. StarNet adheres to a traditional layered network structure, comprising four stages, with the number of channels doubling at each stage and a channel expansion factor of four. Convolutional layers are utilized for downsampling, and feature extraction is performed using Star Blocks, which incorporate deep convolution at the final stage. Batch normalization, rather than layer normalization, follows the deep convolution layer to enhance computational efficiency. The network structure iteratively employs multiple star-shaped blocks for feature extraction, eschewing complex structures or hyperparameter settings.

The essence of StarNet lies in its star-shaped operation method, which adeptly captures high-dimensional and nonlinear feature spaces within low-dimensional realms. In a single-layer neural network, the weights and biases are represented as $W=\left[\begin{array}{l} W\\ B \end{array}\right]$ and $X=\left[\begin{array}{l} X\\ 1 \end{array}\right]$, respectively. The star-shaped operation is defined as ${(W}_{1}^{T}{X)\;*\;(W}_{2}^{T}X)$. For a single output channel and single-element input, we define *w*_1_, *w*_2_, and $x\in {\mathbb{R}}^{(d+1)\times 1}$, where *d* is the input channel number, and for multiple output channels and multi-feature elements $W_{1}{,W}_{2}\in {\mathbb{R}}^{\left(d+1)\times (d^{\prime }+1\right)}$, $X\in {\mathbb{R}}^{(d+1)\times n}$, the star-shaped operation can also be expressed as

(1) $$\begin{array}{ll} w_{1}^{T}x*w_{2}^{T}x & =\left(\sum_{i=1}^{d+1}w_{1}^{i}x^{i}\right)*\left(\sum_{j=1}^{d+1}w_{2}^{j}x^{j}\right)\\ & =\sum_{i=1}^{d+1}\sum_{j=1}^{d+1}w_{1}^{i}w_{2}^{j}x^{i}x^{j}\\ & =\underset{(d+2)(d+1)/2}{\underbrace{\alpha_{\left(1,1\right)}x^{1}x^{1}+\cdots +\alpha_{\left(4,5\right)}x^{4}x^{5}+\cdots +\alpha_{\left(d+1,d+1\right)}x^{d+1}x^{d+1}}} \end{array}$$

where *i* and *j* are the index channel numbers, and α is each coefficient:

(2) $$\alpha (i,j)=\left\{ \begin{array}{ll} w_{1}^{i}w_{2}^{j} & \mathrm{if}\;i==j\\ w_{1}^{i}w_{2}^{j}+w_{1}^{j}w_{2}^{i} & \mathrm{if}\;i!=j \end{array}\right.$$

From Equation (1), it can be seen that except for the $\alpha_{\left(d+1,:\right)}x^{\left(d+1\right)}x$ term, the rest are non-linear with *x*. Therefore, when performing star-shaped operations in d-dimensional space, it is represented in (d+2)(d+1)/2 hidden dimensional space without generating any additional calculations within a single layer, significantly amplifying the feature dimensions and demonstrating the ability to achieve high-dimensional performance operations.

#### 2.2.2. C2f-ELC Network

**Figure 5:**
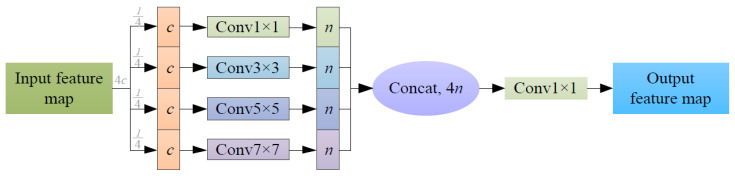
ELC convolution module.

Given the limitations of mobile hardware devices for field operations, the detection algorithm must have minimal parameters, real-time detection capabilities, and sufficient accuracy in detecting safflower filaments in unstructured scenes. In response to these requirements, this study introduces a novel ELC convolution module, as depicted in Figure 5. This structure is inspired primarily by the design principles of GhostNet [25], addressing the issue of large parameters and computational overhead associated with standard convolution by utilizing half of the original input features. It generates the same number of features as standard convolution through simple linear operations (inexpensive operations), thereby resolving the problem of feature redundancy. Specifically, the ELC convolution module groups the input features and processes each group with convolution kernels of varying sizes, optimizing the parameter count and generating multi-scale feature information. However, each group of feature channels remains independent, and inter-group information exchange is not possible. To address this, the design incorporates the pointwise convolution method proposed by MobileNet [23], enabling interaction between the features of each group and significantly reducing computational load and complexity.

As illustrated in Figure 5, the ELC convolution module divides the input features into 4 groups of features with channel number *c*, then processes the features with 1 × 1, 3 × 3, 5 × 5, and 7 × 7 convolutions to obtain 4 groups of features with channel number *n* at different scales, and then integrates the information of each group through 1 × 1 convolution, keeping the output channel number equal to the input channel number. Assuming *C*_1_ is the computational volume of ordinary convolution, and *C*_2_ is the computational volume of the ELC convolution module, it can be seen from Equations (3) and (4) that the computational volume of ordinary convolution with kernel size 3 is 1.7 times that of the ELC convolution module.

(3) $$C_{1}=k\cdot k\cdot 4c\cdot 4n\cdot x\cdot y$$

(4) $$C_{2}=(k_{1}^{2}+k_{3}^{2}+k_{5}^{2}+k_{7}^{2})c\cdot n\cdot x\cdot y$$

In the equations, *k* represents the convolution kernel size; *c* represents the input feature channel number; *n* represents the output feature channel number; *x* and *y* represent the output feature map size; and *k*_1_, *k*_3_, *k*_5_, and *k*_7_ represent the convolution kernels of 1 × 1, 3 × 3, 5 × 5, and 7 × 7, respectively.

**Figure 6:**
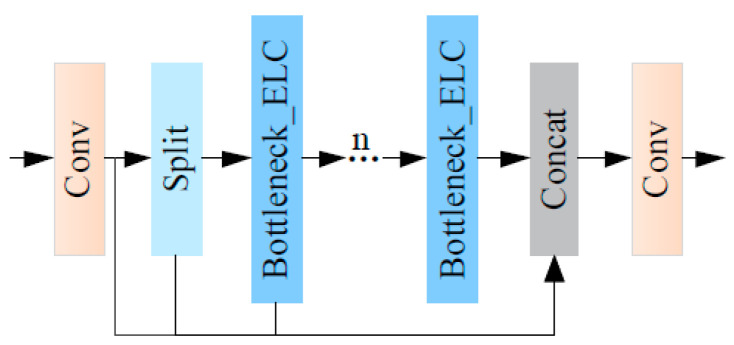
C2f_ELC module.

**Figure 7:**
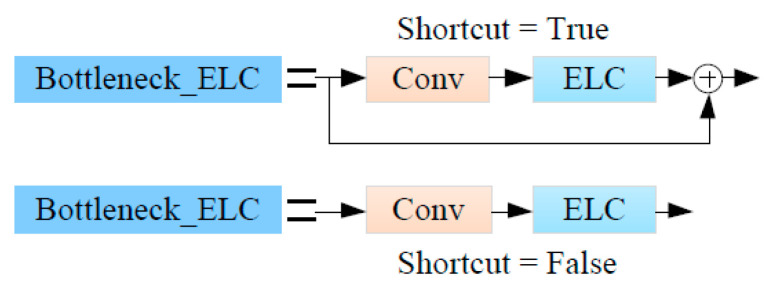
Bottleneck_ELC convolution module.

The C2f_ELC network structure is shown in Figure 6. The ELC convolution module is used to improve the Bottleneck in C2f by replacing the second convolution in its Bottleneck module with ELC convolution, and the improved module is named Bottleneck_ELC, as shown in Figure 7.

#### 2.2.3. Detect_EL Detection Head

**Figure 8:**
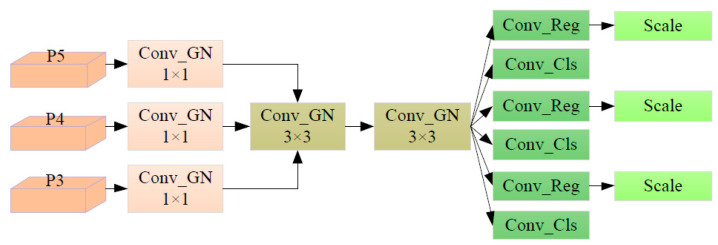
Detect_EL detection module.

The detection of safflower filaments mainly operates in unstructured planting environments with low edge device computing power, requiring the detection model to have as few parameters as possible. The original YOLOv8 detection head requires two 3 × 3 convolutions and one 1 × 1 convolution to extract information for each head, resulting in a large number of model parameters and a traditional single-scale prediction structure, which performs poorly on small target safflower filaments obstructed in the field. Based on this, this study refers to the FCOS paper [28], which has demonstrated the use of GN (GroupNorm) to enhance the performance of the detection head in positioning and classification, and proposes a lightweight shared convolution detection head, Detect_EL, as shown In Figure 8. By using shared convolution, redundant calculations are reduced, computational efficiency is improved, and the model becomes more suitable for deployment on mobile edge devices. While using shared convolution, a Scale layer Is used to scale the output features to address the Issue of Inconsistent target scales detected by each detection head, minimizing accuracy loss under conditions of fewer parameters and lower computational volume, making it significantly advantageous for real-time detection of small unstructured safflower filament targets in low-power field devices.

#### 2.2.4. PioUv2 Loss Function

The default CioU loss function in YOLOv8 mainly measures the overlap between the predicted and true bounding boxes, which is a comprehensive performance indicator considering the overlap ratio, center point distance, and aspect ratio. When the aspect ratio of the predicted bounding box and the true bounding box are linearly related, the CioU loss function degenerates into the IoU loss function, resulting in poor generalization and slow convergence speed for detecting small unstructured safflower filament targets in the field, and it cannot fully reflect the differences and degradation between bounding boxes. To overcome the low detection accuracy caused by this defect, this study improves the CioU loss function to the PioUv2 loss function. PioUv2 is obtained by adding an attention layer to the PioU loss, further enhancing the model’s convergence speed and detection accuracy. Its calculation formula is as follows:

(5) $$f(x)=1-e^{-x^{2}}$$

(6) PIoU=IoU−f(P),−1≤PIoU≤1

(7) $$L_{PIoU}=1-PIoU=L_{IoU}+f(P),\;0\leq L_{PIoU}\leq 2$$

(8) $$q=e^{-p},q\in (0,1]$$

(9) $$u(x)=3x\cdot e^{-x^{2}}$$

(10) $$L_{PIoU_{-}v2}=u(\lambda q)\cdot L_{PIoU}=3\cdot (\lambda q)\cdot e^{-{\left(\lambda q\right)}^{2}}\cdot L_{PIoU}$$

In the formula, u(λq) represents the attention function, and *q* replaces the penalty factor *P*; when *q* equals 1, *P* equals 0, indicating complete overlap between the predicted and true bounding boxes. λ is a hyperparameter controlling the attention function. In PioUv2, there is only one hyperparameter λ, greatly simplifying the tuning process, with λ set to 1.1 in this improvement.

### 2.3. Experimental Environment Configuration and Training Strategy

**Figure 9:**
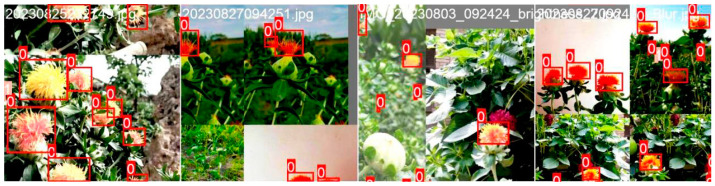
Mosaic data augmentation strategy.

In this experiment, the training environment used a Windows 11 operating system with hardware configuration of 384 GB memory and an AMD Ryzen Threadripper PRO 3975WX 32-Core processor (AMD, Santa Clara, CA, USA). The required libraries for the model configuration include Anaconda 3.8, Python 3.8, OpenCV, CUDA 11.6, and Pytorch 1.13.1 deep learning framework. To increase the diversity of the dataset and its impact on model performance, Mosaic data augmentation was used during training. This method integrates four different photos through rotation, translation, scaling, and cropping to form new data images, as shown in Figure 9. Specific training strategy parameters are listed in Table 1.

### 2.4. Performance Evaluation Metrics

The objective of this study is to minimize model parameters, computational load, model size, and detection time while maintaining the detection accuracy of safflower filaments. Therefore, seven metrics were selected for evaluation: Parameter, Floating Point Operations (FLOPs), Precision, Recall, Mean Average Precision (mAP@0.5), Weights, and Frames Per Second (FPS).

## 3. Results and Analysis

### 3.1. Model Training

**Figure 10:**
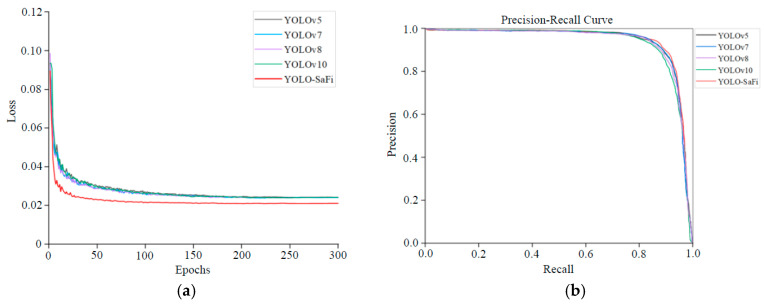
Curves of different model training. (**a**) Loss curve. (**b**) P-R curve.

The training was set for 300 epochs, and the program terminated automatically when the mean average precision showed no significant increase. The training process is shown in Figure 10a,b. From the Loss training curve in Figure 10a, it is evident that the learning rate was high in the early training stages, with the Loss curve converging rapidly. At around 250 epochs, the Loss stabilized at 0.025. Compared to other baseline models, the YOLO-SaFi model converged the fastest and had the lowest stable Loss value. Figure 10b shows the P-R curve for different models. The larger the area enclosed by the P-R curve and the coordinate axes, the better the model performance. The proposed YOLO-SaFi algorithm’s P-R curve (indicated in red) is closer to the upper right corner, with the largest enclosed area, indicating high stability and fitting performance. The final mAP reached 93.9%, and the recall rate was 88.6%.

### 3.2. Ablation Experiment

To verify the effectiveness of each module in the improved algorithm for recognizing safflower filaments, YOLOv8n was used as the baseline model. Four improved algorithms (StarNet, C2f_ELC, Detect_EL, PIoUv2) were introduced sequentially in various combinations for ablation experiments. The results are shown in Table 2.

The ablation results in Table 2 indicate that, compared to the YOLOv8n baseline model, the YOLOv8n + StarNet model reduced parameters, computation, precision, and weight size by 26.7%, 19.8%, 1.0%, and 25.5%, respectively, while increasing recall and detection speed by 0.5% and 56.1 fps, demonstrating the effectiveness of the lightweight backbone network improvement. The YOLOv8n + C2f_ELC model showed decreases in parameters, computation, and weight size with increases in recall and mAP, indicating that the proposed ELC convolution module enhances the model’s multi-scale feature learning expression capability and is more suitable for lightweight applications than regular convolution. The YOLOv8n + Detect_EL model reduced parameters, computation, and weight size by 23.3%, 19.8%, and 21.1%, respectively, while improving recall and mAP by 1.5 and 0.2 percentage points. The YOLOv8n + PIoUv2 model improved precision, recall, mAP, and detection speed, demonstrating the advantages of the improved PIoUv2 loss function in detection accuracy and speed.

In summary, although individual improved modules have performance parameter advantages, issues such as a slight decrease in mAP after changing the Backbone exist. To address this, a multi-module improvement strategy was proposed, synchronously improving the Neck layer, Head layer, and Loss function while improving the Backbone network. For example, compared to the YOLOv8n baseline model, the YOLOv8n + StarNet + C2f_ELC + Detect_EL model reduced parameters, computation, precision, and weight size by 50%, 40.7%, 0.4%, and 48.2%, respectively, while increasing recall, mAP, and detection speed by 1.4%, 0.2%, and 29.3 fps. Further improving the loss function of the YOLOv8n + StarNet + C2f_ELC + Detect_EL model to PIoUv2 and naming it the YOLO-SaFi model, compared to the baseline YOLOv8n, reduced parameters from 3.0 × 10^6^ to 1.5 × 10^6^, computation from 8.1 × 10^9^ to 4.8 × 10^9^, and weight size from 5.96 MB to 3.09 MB, while increasing precision, recall, mAP@0.5, and detection speed by 0.3%, 1.9%, 0.3%, and 88.4 fps, respectively.

### 3.3. Baseline Model Comparison Experiment

To further verify the detection effect of the improved model on safflower filaments in unstructured environments, the improved YOLO-SaFi was compared with the lightweight models of Fast R-CNN, SSD, YOLOv3, YOLOv5, YOLOv6, YOLOv7, YOLOv8, YOLOv9, and YOLOv10 under the same conditions. The evaluation metrics were Parameter, FLOPs, mAP@0.5, and FPS, and the results are shown in Table 3.

The results in Table 3 show that YOLO-SaFi outperformed SSD, YOLOv3, YOLOv5, and YOLOv8 baseline models in parameters, computation, mAP, and detection speed. Compared to SSD, YOLO-SaFi reduced parameters and computation by 93.6% and 98.2%, respectively, while increasing mAP and inference speed by 0.5% and 266.1 fps. Compared to YOLOv3, parameters and computation were reduced by 87.6% and 74.6%, respectively, while increasing mAP and inference speed by 2.5% and 41.8 fps. Compared to YOLOv5, parameters and computation were reduced by 40.0% and 32.4%, respectively, while mAP and inference speed increased by 0.8% and 10.2 fps. Compared to YOLOv8, parameters and computation were reduced by 50.0% and 44.4%, respectively, while mAP and inference speed increased by 0.1% and 88.4 fps.

Comparing YOLO-SaFi with Fast R-CNN, YOLOv7, and YOLOv9 baseline models, although the improved model’s mAP decreased by 0.8%, 0.2%, and 1.1%, parameters were reduced by 98.9%, 75%, and 97.8%, and computation was reduced by 98.7%, 63.6%, and 98.5%, respectively, with inference speeds increasing by 303.9 fps, 166.2 fps, and 313.3 fps, respectively. Compared to YOLOv6 and YOLOv10 baseline models, although detection speed slightly decreased, parameters, computation, and mAP showed significant improvements. The results indicate that the proposed YOLO-SaFi model has a clear advantage over existing baseline models, capable of significantly reducing parameters and computation while maintaining slight fluctuations in detection accuracy and speed, making it more suitable for deployment on edge devices for field safflower filament harvesting robots.

### 3.4. Model Detection Effect Comparison in Different Scenes

**Figure 11:**
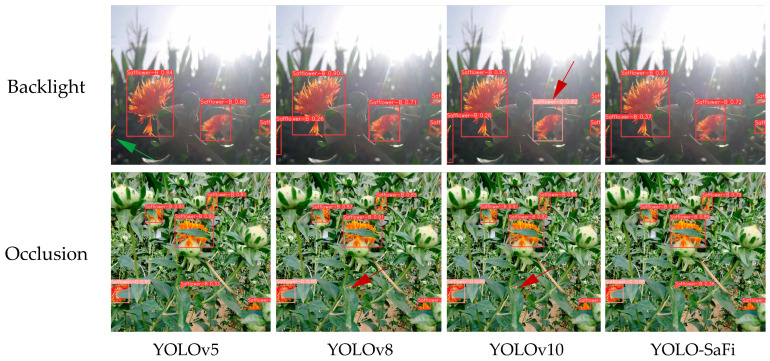
Detection effect of different scenarios.

Detecting safflower filaments in backlight and occlusion scenarios is challenging. To further verify the improved YOLO-SaFi model’s generalization performance for small targets in multi-scene unstructured environments, 100 images of safflower filaments under backlight and occlusion scenarios were randomly selected. Using weight files, recall, and mAP as evaluation metrics, a comparative analysis was conducted on the detection performance of the most commonly used YOLOv5, YOLOv8, and the latest YOLOv10 baseline models in three scenarios. The detection results are shown in Table 4, and the detection effects are shown in Figure 11.

The comparative analysis in Table 4 shows that the improved model proposed in this study has the smallest memory footprint and outperforms YOLOv5, YOLOv8, and the latest YOLOv10 models in backlight and occlusion scenarios. However, all models have lower recall and mAP, especially under occlusion conditions, with the maximum precision at only 81.3%, recall at only 76.9%, and mAP at only 80.4%.

Figure 11 shows that in the backlight scenario, the YOLOv5 model had missed detections (indicated by the green arrows), and the YOLOv10 model had false detections (indicated by the red arrows). The YOLOv8 and the improved YOLO-SaFi had better detection results. Under occlusion conditions, both YOLOv8 and YOLOv10 exhibited missed detections of small safflower filament targets (indicated by the red arrows), while YOLOv5 and the improved YOLO-SaFi had better detection results. The main reasons for missed and false detections are the small size of the safflower filament targets, reduced brightness of blooming filaments due to light effects, severe occlusion by branches and leaves, and the small color difference between the filaments and the background. Therefore, the proposed YOLO-SaFi model, with the smallest parameters and computation, exhibited higher overall detection performance than other state-of-the-art models.

### 3.5. Model Feature Visualization

**Figure 12:**
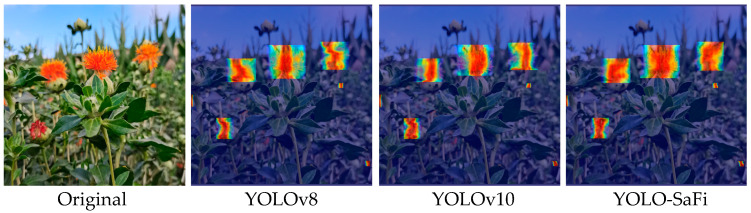
Heatmaps of different models.

To better illustrate the detection performance of the improved Yolo-SaFi model on small safflower filament targets in unstructured environments, this study used Grad-CAM (Gradient-weighted Class Activation Mapping) [29,30] heat map visualization analysis to compare the key regions of heat maps generated by the baseline YOLOv8 and YOLOv10 model. The redder the color, the better the model’s detection performance on small safflower filament targets. The detection results are shown in Figure 12.

Figure 12 shows that compared to the heat map output by the YOLOv8 and YOLOv10 model detection head, the heat map output by the improved YOLO-SaFi model covered a wider area of safflower filaments, with brighter colors, indicating a greater contribution of the safflower filament area to the YOLO-SaFi model’s decision making. This better identifies the characteristics of blooming and declining filaments, effectively demonstrating the rationality and reliability of the improved model proposed in this study, providing a lightweight model for deployment on edge devices for unstructured safflower filament detection in the field.

### 3.6. Edge Device Deployment Verification

**Figure 13:**
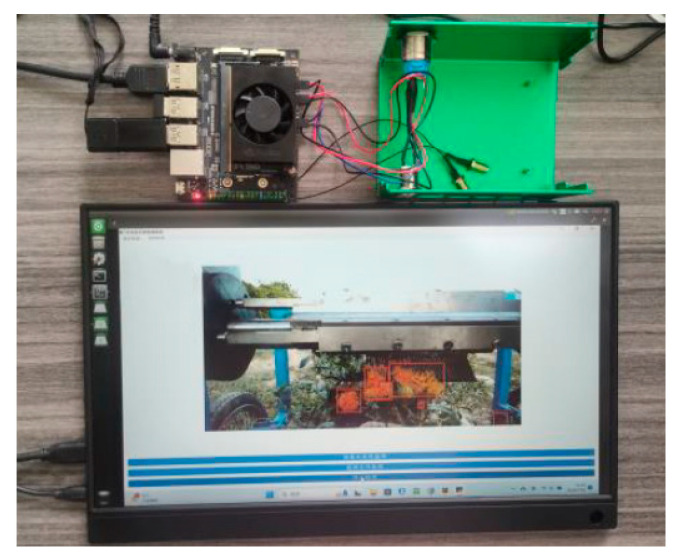
Edge device deployment.

To verify the performance of the improved YOLO-SaFi model on edge devices, this study deployed the YOLO-SaFi model on the NVIDIA Jetson Orin Nano 8 GB device (NVIDIA, Santa Clara, CA, USA), which has 1024 CUDA cores and 32 Tensor Cores. As shown in Figure 13, the TensorRT acceleration framework was used to provide high inference speeds for the model.

**Figure 14:**
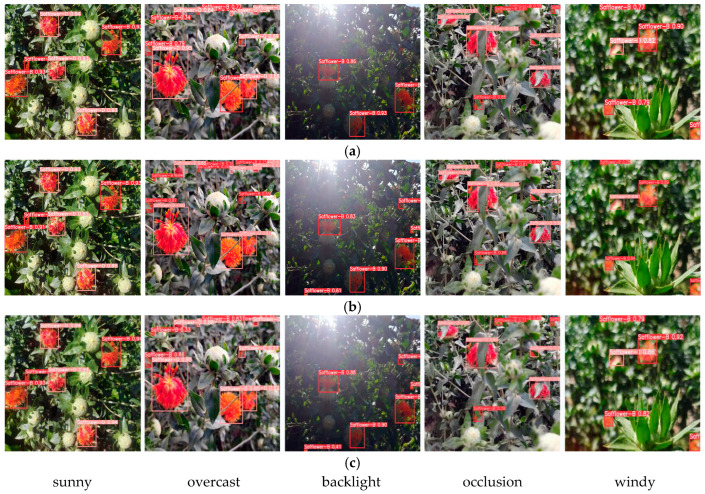
Detection effects of different model deployments. (**a**) YOLOv8. (**b**) YOLOv10. (**c**) YOLO-SaFi.

To further verify the detection performance of the improved YOLO-SaFi model on edge devices, this experiment selected safflower filaments in unstructured environments on sunny, overcast, backlight, occlusion, and windy conditions, and it conducted comparative experiments using YOLOv8, YOLOv10, and YOLO-SaFi algorithm models. The detection results are shown in Table 5, and the detection effects are shown in Figure 14. Compared with YOLOv8 and YOLOv10, the improved model YOLO-SaFi has the highest average recognition accuracy and the best confidence in unstructured environments, with no missed or incorrect detections. YOLOv8 missed three small safflower filaments under backlight conditions; under occlusion conditions, both YOLOv8 and YOLOv10 exhibited repeated incorrect detections of multiple detection frames for a single filament, and the confidence was relatively low.

## 4. Discussion

This study addresses the challenges of severe occlusion of safflower filaments, low visual recognition accuracy, and substantial algorithm model size in unstructured environments. It introduces a lightweight YOLO-SaFi network. Compared to the most renowned and advanced networks, this new network enhances parameter count, reduces computational cost, and increases detection speed, demonstrating superior overall detection performance. However, it exhibits lower recall rates and mean average precision in backlit and occluded environments, with a maximum recall rate of only 76.9% and a mean average precision of just 80.4%. Comparative analysis with other advanced recognition algorithms reveals their inferiority to the YOLO-SaFi algorithm. The primary issues are the severe occlusion of branches and filaments in unstructured environments and variable lighting conditions. Under occluded conditions, the model often misidentifies a single filament as multiple filaments, resulting in repeated detections. In backlit conditions, the reduced brightness of the filaments hampers their distinction from the background, causing missed detections. Consequently, future research will focus on enhancing the performance of safflower filament recognition in backlit and occluded scenarios, as well as the coordination control of edge devices and the Delta robotic arm of intelligent harvesting robots, aiming to achieve more lightweight and efficient intelligent safflower filament harvesting.

## 5. Conclusions

To achieve rapid and accurate detection of safflower filaments in unstructured environments, this study proposes a lightweight YOLO-SaFi algorithm model, derived from the YOLOv8n model. The Backbone, Neck, Head, and Loss function components were redesigned, leading to the following conclusions:

(1) To reduce model parameters and computational cost, the lightweight StarNet network was introduced in the Backbone network for redesigning, a new ELC convolution module was proposed in the Neck network to redesign the C2f module, a new lightweight shared convolution detection head Detect_EL was designed in the Head layer, and CIoU was improved to PIoUv2 in the loss function. Compared with the YOLOv8 baseline model, the optimized YOLO-SaFi model reduced parameters, computational cost, and weight file size by 50.0%, 40.7%, and 48.2%, respectively, while improving precision, recall, mAP@0.5, and detection speed by 0.3%, 1.9%, 0.3%, and 88.4 f/s, respectively.

(2) To verify the detection effect of the improved YOLO-SaFi model, this study conducted performance comparison experiments with lightweight models of Fast R-CNN, SSD, YOLOv3, YOLOv5, YOLOv6, YOLOv7, YOLOv8, YOLOv9, and YOLOv10 in the same environment. The experimental results showed that the YOLO-SaFi model has advantages in parameter count, computational cost, weight size, and mean average precision, making it more suitable for deployment on field mobile edge devices.

(3) To further verify the generalization performance of the improved YOLO-SaFi algorithm on small safflower filament targets, detection performance was compared and analyzed in backlit and occluded scene datasets with YOLOv5, YOLOv8, YOLOv10, and YOLO-SaFi. The results showed that the YOLO-SaFi algorithm outperformed YOLOv5, YOLOv8, and the latest YOLOv10 models in terms of comprehensive performance in backlit and occluded scenes, improving the detection effect of safflower filaments. Heatmap visualization analysis effectively demonstrated the rationality and reliability of the improved algorithm, providing a lightweight model for deployment on safflower filament detection edge devices.

(4) Deploying the improved YOLO-SaFi model on Nvidia Jetson Orin Nano devices further verified the effectiveness of the improved model, meeting the recognition performance requirements of intelligent picking robots for picking safflower filaments in the field.

## Figures and Tables

**Table 1:** Training strategy parameters.

Parameter	Value
Epochs	300
lr0	0.01
Optimizer	SGD
Momentum	0.937
Weight decay	0.0005
Batch	16
Workers	8

**Table 2:** Ablation experiment.

Base Model	StarNet	C2f_ELC	Detect_EL	PIoUv2	Parameter/M	FLOPs/G	P/%	R/%	mAP@0.5 /%	Weights /MB	FPS/ (f·s^−1^)
YOLOv8n	×	×	×	×	3.0	8.1	91.9	86.8	93.6	5.96	256.4
YOLOv8n	√	×	×	×	2.2	6.5	90.9	87.3	93.4	4.44	312.5
YOLOv8n	×	√	×	×	2.9	8.0	92.7	87.6	93.8	5.86	261.6
YOLOv8n	×	×	√	×	2.3	6.5	91.6	88.3	93.8	4.70	287.4
YOLOv8n	×	×	×	√	3.0	8.1	92.4	88.4	94.0	5.96	260.3
YOLOv8n	√	√	×	×	2.1	6.4	92.3	86.1	93.5	4.34	333.3
YOLOv8n	√	×	√	×	1.6	4.9	91.6	88.0	93.8	3.19	294.1
YOLOv8n	√	×	×	√	2.2	6.5	92.1	86.6	93.2	4.44	341.2
YOLOv8n	√	√	√	×	1.5	4.8	91.5	88.2	93.8	3.09	286.3
YOLOv8n	√	√	×	√	2.1	6.4	92.1	87.5	93.6	4.34	277.8
YOLOv8n	√	×	√	√	1.6	4.9	91.4	88.3	93.7	3.2	321.3
YOLOv8n	√	√	√	√	1.5	4.8	92.2	88.6	93.9	3.09	344.8

Note: “×” indicates not added; “√” indicates added.

**Table 3:** Comparative experiments of baseline models.

Model	Parameter/M	FLOPs/G	mAP@0.5/%	FPS/(f·s^−1^)
Fast R-CNN	136.7	369.7	94.7	40.9
SSD	23.6	273.2	93.4	78.7
YOLOv3	12.1	18.9	91.4	303.0
YOLOv5	2.5	7.1	93.1	334.6
YOLOv6	4.2	11.8	92.9	360.4
YOLOv7	6.0	13.2	94.1	178.6
YOLOv8	3.0	8.1	93.6	256.4
YOLOv9	70	314.4	95.0	31.5
YOLOv10	2.7	8.2	93.3	384.6
YOLO-SaFi	1.5	4.8	93.9	344.8

**Table 4:** Comparison results of detection in different scenarios.

Scene	Model	Weights/MB	P/%	R/%	mAP@0.5/%
Backlighting	YOLOv5	5.02	84.3	84.5	90.9
	YOLOv8	5.96	86.7	85.4	91.5
	YOLOv10	5.49	87.2	85.6	91.2
	YOLO-SaFi	3.09	86.9	85.6	91.7
Occlusion	YOLOv5	5.02	79.9	75.3	79.3
	YOLOv8	5.96	78.6	75.5	79.3
	YOLOv10	5.49	82.1	68.2	77.0
	YOLO-SaFi	3.09	81.3	76.9	80.4

**Table 5:** Comparison of parameters for device deployment of different models.

Model	P/%	R/%	mAP@0.5/%	FPS/(f·s^−1^)
YOLOv8	89.6	84.0	90.8	26.3
YOLOv10	90.6	83.8	90.6	29.4
YOLO-SaFi	90.9	84.5	91.9	29.1

## Data Availability

The data in the paper is presented in the form of charts and graphs. For more detailed data, please obtain it from the corresponding author.
